# Impact of repeated *in-vitro* bacterial culture on virulence and antibiotic resistance characteristics: a study of Gram-positive and Gram-negative fish pathogens

**DOI:** 10.3389/fmicb.2025.1601681

**Published:** 2025-05-21

**Authors:** Angana Bag, Vikash Kumar, Anupam Adhikari, Biswajit Mandal, Souvik Dhar, Basanta Kumar Das

**Affiliations:** Aquatic Environmental Biotechnology and Nanotechnology (AEBN) Division, ICAR-Central Inland Fisheries Research Institute (CIFRI), Barrackpore, India

**Keywords:** *in-vitro* subculturing, virulence, antimicrobial resistance, fish pathogens, aquaculture

## Abstract

The ability of bacteria to respond to environmental changes is critical for survival. This enables them to withstand stress, form complex communities, and trigger virulence responses during host infections. In this study, we examined the effects of repeated *in vitro* subculturing on the virulence and antimicrobial resistance (AMR) profiles of Gram-negative and Gram-positive fish pathogens. The fish pathogenic bacterial isolates, namely *Lactococcus lactis*, *Enterococcus gallinarum*, *Proteus penneri*, and *Escherichia coli*, underwent 56 consecutive subcultures in tryptic soy broth and were evaluated for virulence, antimicrobial susceptibility, and AMR gene expression. The results revealed a significant decrease in the virulence of Gram-positive pathogens. Both *L. lactis* and *E. gallinarum* exhibited a marked reduction in the mortality rates of *Labeo rohita* after repeated subculturing, ultimately achieving 0% mortality by day 56. This suggests losing key virulence factors, such as toxins and adhesins, under non-selective conditions. In contrast, Gram-negative bacteria, particularly *P. penneri* and *E. coli*, exhibited higher levels of virulence throughout the study, even though mortality rates gradually declined. The antimicrobial resistance profiles of *L. lactis* remained steady, demonstrating consistent resistance to a wide range of antibiotics, including rifampicin and polymyxin B. Meanwhile, *E. gallinarum* showed slight variations in resistance, especially to colistin, while *P. penneri* and *E. coli* experienced changes in resistance to multiple antibiotics, including polymyxin B and tetracycline, after 42 days of subculturing. Importantly, no genetic alterations were detected in AMR-related genes through quantitative PCR analysis, indicating that the observed changes in resistance were likely phenotypic rather than genetic. This study underscores the critical need for ongoing surveillance in aquaculture pathogen management, emphasizing the dynamic nature of bacterial virulence and resistance profiles that can develop from prolonged subculturing.

## 1 Introduction

Aquaculture is a rapidly expanding industry essential for meeting global seafood demands. However, the intensification of fish farming has increased the prevalence of antibiotic resistance, posing significant threats to industry sustainability and public health (Samanta and Bandyopadhyay, 2019; Milijasevic et al., 2024; Ljubojević Pelić et al., 2024). The extensive and often indiscriminate use of antibiotics in aquaculture to combat bacterial infections has been identified as a major driver of antimicrobial resistance (AMR), facilitating the selection and spread of resistant bacteria (Paul, 2024; Embeti et al., 2023). These resistant pathogens pose a dual threat by compromising fish health and increasing the potential for zoonotic transmission to humans, emphasizing the critical need for monitoring and mitigation strategies (Martínez and Baquero, 2002; Samanta and Bandyopadhyay, 2019). Globally, AMR has emerged as a pressing public health issue. It is estimated that bacterial AMR directly caused 1.27 million deaths and contributed to 4.95 million deaths in 2019. The World Health Organization (WHO) projects that AMR could lead to 350 million deaths by 2050 if not addressed, highlighting the urgent need for collective global action, including international treaties to combat this crisis. Alarmingly, the 2022 Global Antimicrobial Resistance and Use Surveillance System (GLASS) report has documented high resistance rates in common bacterial infections, underscoring the diminishing effectiveness of standard antibiotics. The evolution of antimicrobial resistance (AMR) is intricately associated with bacterial pathogenicity due to genetic adaptations. These adaptations include mechanisms such as horizontal gene transfer, as well as co-evolution with mobile genetic elements, including plasmids.

Bacteria evolve mechanisms that underlie the changes in external stress. Bacterial cells respond to stress at two different levels, including protein activity, which is an immediate response, and gene transcription, which is a long-term response. Microbial phenotypes, shaped by environmental feedback and evolutionary trade-offs (Zhu and Dai, 2024; Caporale, 2024), undergo significant alterations under laboratory conditions (López-Maury et al., 2008). Subculturing exerts selective pressures that may diminish pathogenicity while concurrently fostering the evolution of antimicrobial resistance (Baquero et al., 2021; Hughes and Andersson, 2017). This adaptation, an important strategy for enhancing stress tolerance in microbial cell factories, involves gradual modifications of microorganisms in a stressful environment to enhance their tolerance. During adaptation, microorganisms use different mechanisms to enhance non-preferred substrate utilization and stress tolerance, thereby improving their ability to adapt for growth and survival. Gram-positive and Gram-negative fish pathogens, which are essential for aquaculture health, exhibit dynamic adaptations to environmental stimuli, impacting virulence factors and resistance mechanisms (Mekalanos, 1992; Smits et al., 2006). These alterations can profoundly influence disease management strategies within aquaculture, thus presenting challenges to the efficacy of therapeutic interventions (Christaki et al., 2020; Bakkeren et al., 2020).

These genetic mechanisms facilitate the development of virulence factors and multidrug-resistant strains (Braz et al., 2020). Plasmids play a crucial role in the dissemination of resistance and virulence genes, thereby contributing to the emergence of highly virulent, multidrug-resistant clones (Beceiro et al., 2013; Carattoli, 2013; Mathers et al., 2015). Additionally, the formation of biofilms and the presence of multidrug efflux pumps enhance bacterial tolerance to antibiotics and influence host-pathogen interactions (Giraud et al., 2017). It is noteworthy that repeated subculturing also influences bacterial AMR profiles via selective pressure and spontaneous mutations. Bacteria that are exposed to antibiotics during subcultures exhibit reduced zones of inhibition, indicating increased resistance (Eagle, 1952; Shamsuddeen and Mustapha, 2017). Conversely, subculturing without antibiotics may result in the loss of heteroresistant subpopulations, consequently altering antibiograms (Metcalfe et al., 2017). The co-evolution of bacterial genomes with plasmids and horizontal gene transfer further enables pathogens such as *E. coli* to adapt to novel metabolic niches, thereby enhancing their virulence potential (Frenkel et al., 2021; Braz et al., 2020).

Consequently, comprehending these dynamics is imperative for formulating innovative strategies aimed at mitigating AMR and effectively combating bacterial virulence. This study conducts an investigation into the effects of repeated *in vitro* subculturing on the phenotypic and genotypic profiles of Gram-negative *Proteus penneri* and *Escherichia coli*, as well as Gram-positive *Lactococcus lactis* and *Enterococcus gallinarum*. Subculturing under controlled conditions has the potential to induce selective pressures, leading to dynamic alterations in virulence factors and resistance patterns. Through the examination of these adaptations, we seek to elucidate bacterial resilience mechanisms and their ramifications for aquaculture health management, thereby contributing to the development of strategies aimed at curbing the proliferation of multidrug-resistant (MDR) pathogens.

## 2 Materials and methods

### 2.1 Bacterial strain collection and subculture

Four bacterial strains representing the Gram-positive bacteria *Lactococcus lactis* (GenBank Accession Number: OR999571) were isolated from *Labeo bata*. *Enterococcus gallinarum* (GenBank Accession Number: OR999570) was isolated from *Hypophthalmichthys nobilis*, while the Gram-negative bacteria *Proteus penneri* (GenBank Accession Number: OP554277) were isolated from *Labeo catla*, and *Escherichia coli* (GenBank Accession Number: OR794370) was isolated from *Carassius auratus*. For bacterial screening from the infected fish, the fish with clinical signs and lesions on the body was sacrificed by using Clove oil as anesthetic (Dabur, India) at 50 μL per liter of water. All strains were collected from the ICAR-CIFRI biotechnology lab. The freshly isolated bacterial strains were subcultured in tryptic soy broth (TSB) media (Hi-Media, Mumbai, Maharashtra, India) and incubated for 24 h at 28°C. These four bacterial strains underwent sequential sub-culturing 56 times under the same culture conditions. During each subculture, the bacterial strains were streaked onto TSA plates to ensure that the culture was not contaminated.

### 2.2 Fish challenge assay

From a nearby hatchery, a healthy *Labeo rohita* fingerling measuring 115.52 ± 2.16 mm in length and weighing 20.26 ± 1.02 g was obtained. Following this, the fish were acclimatized for 2 weeks in 200-liter fiber-reinforced plastic (FRP) tanks. They were fed suitable commercial floating feed (crude protein: 30%, crude lipid: 5%), with 3–5% of their body weight provided twice daily. The photoperiod during the culture period was maintained with a 12-h light and a 12-h dark cycle, along with aeration (DO 6.8–7.2) and a controlled temperature chamber that kept the water at 27.5–28.5°C. After this procedure, the fish were used for challenge studies. For the challenge investigation, a sterile 15 mL Falcon tube containing a strain of bacteria was incubated for 24 h at 28°C in tryptic soy broth (TSB) medium (Hi-Media). Challenge studies were conducted every 7 days. The McFarland Standards technique was utilized to determine the concentration of bacteria. The bacterial cell pellet was recovered by centrifugation at 8,000 rpm for 8 min and rinsed with phosphate-buffered saline, pH 7.4 (HIMEDIA, India). Ten fish were used for each bacterium challenge investigation, and the experimental fish were intraperitoneally injected with 200 μL (10^7^ colony-forming units/ml) of the bacterial solution. The fish were maintained in an FRP tank and monitored every 48 h. Water quality parameters throughout the experimental trial included pH, dissolved oxygen, and alkalinity, which measured 7.30 ± 0.05, 5.3 ± 0.30 mg/L, and 78.0 ± 1.4 mg/L, respectively. The water temperature was maintained at 28°C.

### 2.3 Antibiogram assay

Antibiotic discs (Hi-Media, Mumbai, Maharashtra, India) were used in the disc diffusion technique (Bauer et al., 1966) to evaluate antibiotic susceptibility, conducted every seventh day to identify the bacteria’s antimicrobial resistance profile under *in vitro* conditions. For this experiment, 25 different antibiotic discs (6 mm in diameter) were used in quintuplicate: Ampicillin (25 μg), Tetracycline (10 μg), Erythromycin (10 μg), Dicloxacillin (1 μg), Streptomycin (254 μg), Doxycycline (10 μg), Ofloxacin (2 μg), Amoxicillin (30 μg), Ceftazidime (30 μg), Cefixime (5 μg), Rifampicin (5 μg), Nalidixic acid (30 μg), Piperacillin (10 μg), Chloramphenicol (30 μg), Polymyxin B (300 μg), Colistin (10 μg), Imipenem (10 μg), Trimethoprim (5 μg), Ciprofloxacin (5 μg), Netilmicin sulfate (30 μg), Tobramycin (10 μg), Cefepime (30 μg), Nitrofurantoin (200 μg), Gentamicin (10 μg), and Fosfomycin (200 μg). Using a plate spreader, 100 μL of pure bacterial culture (5.8 × 10^8^ CFU/mL) was applied to each TSA plate, and the overnight-grown bacterial culture was counted. On a single plate, five or six distinct antibiotic discs were placed. Afterward, the agar plates were covered with parafilm and incubated at 28°C for 24 h. The Clinical and Laboratory Standards Institute guidelines were followed to calculate the millimeter diameters of the inhibitory halos surrounding the antibiotic discs. Sensitive, moderate, and resistant outcomes were observed.

### 2.4 RNA isolation and cDNA synthesis

Four newly cultivated bacterial suspensions were used for RNA isolation every 7 days. A bacterial pellet was formed by centrifuging at 10,000 rpm for 6 min. This process was repeated several times until the desired quantity of pellets was obtained. The CARBUnn MagXene RNA Extraction Kit (Magnetic Beads Based) (JlTM C Genes Pvt. Ltd) (Gautam Buddha Nagar, U.P.-2013301, India) is utilized for RNA isolation. The quantity and quality of the RNA were assessed using a NanoDrop spectrophotometer (Thermo Scientific India) at an absorbance of 260/280 nm after it had been treated with DNase I (RNase free; Thermo Scientific) to remove genomic DNA contamination. A 1% agarose gel was employed to verify the integrity of the RNA. Subsequently, complementary DNA (cDNA) was synthesized through reverse transcription using the RevertAidTM H Minus First Strand Synthesis Kit (Thermo Scientific India). One microliter of random hexamer primer solution was combined with 1 microgram of total RNA. Following this, 8 microliters of the reaction mixture were added, which included 20 units of ribonuclease inhibitor, 200 units of RevertAid™ H minus M-MuLV reverse transcriptase, and 4 microliters of 5x reaction buffer (0.25 mol/L Tris–HCl pH 8.3, 0.25 mol/L MgCl2, 0.05 mol/L DTT), along with 2 microliters of 0.01 mol/L dNTP mix. After a 5-min incubation at 25°C, the reaction mixture was incubated for 60 min at 42°C. The process was stopped by heating to 70°C for 5 min and then cooling to 4°C.

### 2.5 Anti-microbial resistance gene expression study

The expression of antibiotic-resistant genes (*sul1, sul2, tetM, tetW, qepA, qnrS, oqxA, oqxB, aac, ami, blaP*) and *ß-actin* (124 bp), a housekeeping gene to check the integrity of RNA, was measured by RT-qPCR using a pair of specific primers with the StepOnePlus Real-time PCR system (Applied Biosystems) (Kumar et al., 2022; Roy et al., 2019; Kumar et al., 2023). The amplification was carried out in a total volume of 20 μL, which included 10 μL of 2X Maxima SYBR Green/ROX qPCR Master Mix (ThermoFisher Scientific), 1 μL of cDNA (50 ng), 8 μL of nuclease-free water, and 0.5 μL of each specific primer (Table 1). Master mixes were prepared for each biological replicate of the sample in triplicate, and RT-qPCR for target and reference genes was conducted with a four-step amplification protocol: initial denaturation (10 min at 95°C); 40 cycles of amplification and quantification (15 s at 95°C, 30 s at 60°C, and 30 s at 72°C); a melting curve (55–95°C) with a heating rate of 0.10°C/s and continuous fluorescence measurement; and cooling (4°C). A negative control reaction was included for each primer set by omitting template cDNA. The comparative CT method (2^−ΔΔCt^ method), as described by Livak and Schmittgen (2001), was used to analyze the expression levels of the target genes, and this was verified using the Pfaffl relative standard curve method (Pfaffl et al., 2002). The log-transformed 2^−ΔΔCt^ value was analyzed using a t-test, and *p* values smaller than 0.05 were considered statistically significant (Table 2).

**Table 1:** List of primers used in the study.

Genes	Primers for qPCR	Amplicon size	Annealing temperature	References
sul1 (Sulfonamide)	CGCACCGGAAACATCGCTGCAC	163 bp	55°C	Pei et al. (2006)
	TGAAGTTCCGCCGCAAGGCTCG			
sul2 (Sulfonamide)	TCCGGTGGAGGCCGGTATCTGG	191 bp	55°C	Pei et al. (2006)
	CGGGAATGCCATCTGCCTTGAG			
tetM (Tetracycline)	ACAGAAAGCTTATTATATAAC	171 bp	55°C	Aminov et al. (2001)
	TGGCGTGTCTATGATGTTCAC			
tetW (Tetracycline)	GAGAGCCTGCTATATGCCAGC	168 bp	55°C	Aminov et al. (2001)
	GGGCGTATCCACAATGTTAAC			
qepA (Quinolone)	CCAGCTCGGCAACTTGATAC	570 bp	55°C	Xia et al. (2010)
	ATGCTCGCCTTCCAGAAAA			
qnrS (Quinolone)	GCAAGTTCATTGAACAGGGT	428 bp	60°C	Bach et al. (2006)
	TCTAAACCGTCGAGTTCGGCG			
oqxA (Olaquindox)	CTCGGCGCGATGATGCT	392 bp	55°C	Kim et al. (2009)
	CCACTCTTCACGGGAGACGA			
oqxB (Olaquindox)	TCCTGATCTCCATTAACGCCCA	131 bp	55°C	Park et al. (2012)
	ACCGGAACCCATCTCGATGC			
aac(6′)-Ib (Aminoglycoside)	TTGCGATGCTCTATGAGTGGCTA	482 bp	55°C	Cattoir and Bicêtre (2008)
	CTCGAATGCCTGGCGTGTTT			
ami (Aminoglycoside)	TGATCCCGTAAATGAGTTGAA	465 bp	60°C	Karumathil et al. (2014)
	GCGGGCAAATGTGATGGTA			
blaP (Beta-lactam)	ACACTAGGAGAAGCCATGAA	861 bp	61°C	Karumathil et al. (2014)
	GCATGAGATCAAGACCGATAC			
Cmr (Chloramphenicol)	CTATTTGAATTTGCGGTTTATA TGCACTTACACCGAAATCTTC	650 bp	61°C	Karumathil et al. (2014)

**Table 2:** Comparison of antimicrobial resistance gene expression between the initial and 56th day of subculture for each gene expression in all other strains, normalized accordingly using the 2^−ΔΔCT^ method.

Genes	Function	Gene expression of freshly isolated bacteria				Gene expression of bacteria after 56th days of subculture			
		Gram-positive bacteria		Gram-negative bacteria		Gram-positive bacteria		Gram-negative bacteria	
		LL	EG	PP	EC	LL	EG	PP	EC
sul1 (Sulfonamide)	Sul gene encodes an altered version of the DHPS enzyme. Show resistance against Sulfonamide Antibiotics	1.01 ± 0.2	1.003 ± 0.04	1.001 ± 0.07	1.053 ± 0.14	1.03 ± 0.09	1.03 ± 0.2	1.003 ± 0.06	1.013 ± 0.08
sul2 (Sulfonamide)		1.01 ± 0.04	1.002 ± 0.3	1.002 ± 0.08	1.001 ± 0.05	1.01 ± 0.08	1.039 ± 0.04	1.003 ± 0.1	1.001 ± 0.06
tetM (Tetracycline)	Encodes a ribosomal protection protein that prevents tetracyclines from binding to the ribosome.	1.00 ± 0.21	1.00 ± 0.1	1.00 ± 0.04	1.004 ± 0.05	1.03 ± 0.2	1.037 ± 0.07	1.003 ± 0.2	1.00 ± 0.08
tetW (Tetracycline)		1.03 ± 0.1	1.05 ± 0.04	1.12 ± 0.06	1.005 ± 0.09	1.16 ± 0.2	1.053 ± 0.09	1.25 ± 0.3	1.001 ± 0.07
qepA (Quinolone)	Encode proteins that protect DNA gyrase and topoisomerase IV (targets of fluoroquinolones), preventing antibiotic-induced DNA damage.	1.1 ± 0.2	1.001 ± 0.08	1.001 ± 0.05	1.1 ± 0.08	1.05 ± 0.04	1.026 ± 0.06	1.001 ± 0.07	1.004 ± 0.05
qnrS (Quinolone)	Encode proteins that protect DNA gyrase and topoisomerase IV (targets of fluoroquinolones), preventing antibiotic-induced DNA damage	1.00 ± 0.08	1.001 ± 0.3	1.001 ± 0.04	1.057 ± 0.04	1.03 ± 0.08	1.021 ± 0.03	1.00 ± 0.1	1.001 ± 0.09
oqxA (Olaquindox)	Genes encode components of a multidrug efflux pump system known as the OqxAB efflux pump in bacteria	1.00 ± 0.2	1.002 ± 0.1	1.001 ± 0.05	1.001 ± 0.09	1.05 ± 0.2	1.039 ± 0.08	1.003 ± 0.07	1.022 ± 0.3
oqxB (Olaquindox)		1.00 ± 0.05	1.001 ± 0.04	1.059 ± 0.06	1.001 ± 0.08	1.05 ± 0.07	1.033 ± 0.09	1.023 ± 0.2	1.001 ± 0.08
aac(6′)-Ib (Aminoglycoside)	Encode enzymes (e.g., acetyltransferases, phosphotransferases, nucleotidyltransferases) that chemically modify the antibiotic, reducing its ability to bind to bacterial ribosomes and inhibit protein synthesis.	1.01 ± 0.04	1.00 ± 0.2	1.033 ± 0.08	1.007 ± 0.05	1.02 ± 0.2	1.034 ± 0.01	1.005 ± 0.3	1.002 ± 0.08
ami (Aminoglycoside)		1.00 ± 0.2	1.006 ± 0.08	1.002 ± 0.02	1.001 ± 0.06	1.02 ± 0.07	1.021 ± 0.2	1.047 ± 0.08	1.001 ± 0.04
blaP (Beta-lactam)	These genes produce enzymes (beta-lactamases) that hydrolyze the beta-lactam ring of antibiotics, rendering them inactive.	1.14 ± 0.2	1.05 ± 0.07	1.001 ± 0.08	1.001 ± 0.2	1.03 ± 0.09	1.025 ± 0.05	1.001 ± 0.08	1.01 ± 0.04
Cmr (Chloramphenicol)	Its primary function is to encode a protein that helps the bacterial cell resist the effects of chloramphenicol, an antibiotic that inhibits protein synthesis by targeting the bacterial ribosome.	1.09 ± 0.03	1.064 ± 0.2	1.001 ± 0.08	1.005 ± 0.1	1.03 ± 0.09	1.020 ± 0.3	1.001 ± 0.05	1.007 ± 0.2

LL, *Lactococcus lactis*; EG, *Enterococcus gallinarum*; PP, *Proteus penneri*; EC, *Escherichia coli*; antimicrobial resistance gene of each bacterium compared with the 1st day of isolated bacteria gene, for each gene expression in all other strains was normalized accordingly using the 2^−ΔΔCT^method.

## 3 Results

### 3.1 Challenge assay with Gram-positive bacteria

**Figure 1:**
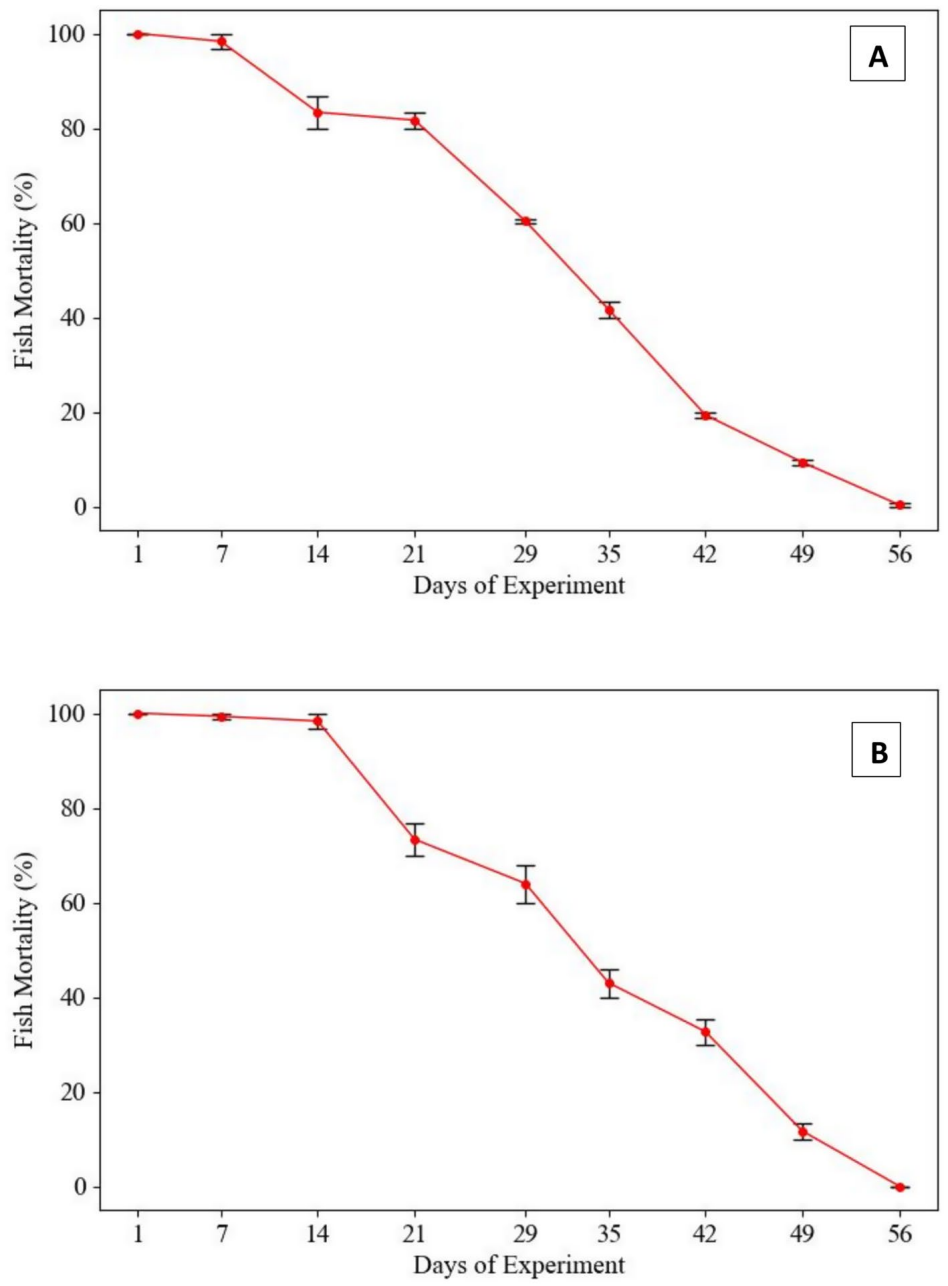
Mortality rates in *Labeo rohita* infected with Gram-positive pathogens following repeated *in-vitro* subculturing. **(A)** *Lactococcus lactis*: Fish mortality progressively declined with each subsequent challenge at 7-day intervals using 1.2 × 10^7^ CFU/fish. Initial mortality was 100% within the first 14 days, with a marked reduction observed thereafter, ultimately reaching 0% by day 56. **(B)** *Enterococcus gallinarum*: Mortality initially plateaued during the first 21 days but gradually decreased, culminating in 0% mortality by day 56. This trend suggests a loss of virulence in both pathogens due to prolonged subculturing in neutral conditions.

To evaluate the host defense mechanism against Gram-positive bacterial pathogens, *Labeo rohita* fingerlings were intraperitoneally injected with 0.2 mL of a bacterial suspension at a final concentration of 1.2 × 10^7^ CFU/fish at weekly intervals. The challenge with *Enterococcus gallinarum* revealed a 100% mortality rate in fish within the first 14 days. However, following subsequent injections of the same bacterial dose, fish mortality declined by day 21, eventually reaching 0% mortality by day 56. A similar trend was observed in fish challenged with *Lactococcus lactis*. A 100% mortality rate was recorded during the first 2 weeks, followed by a gradual decline, culminating in 0% mortality by the 56th day (Figure 1).

### 3.2 Challenge assay with Gram-negative bacteria

**Figure 2:**
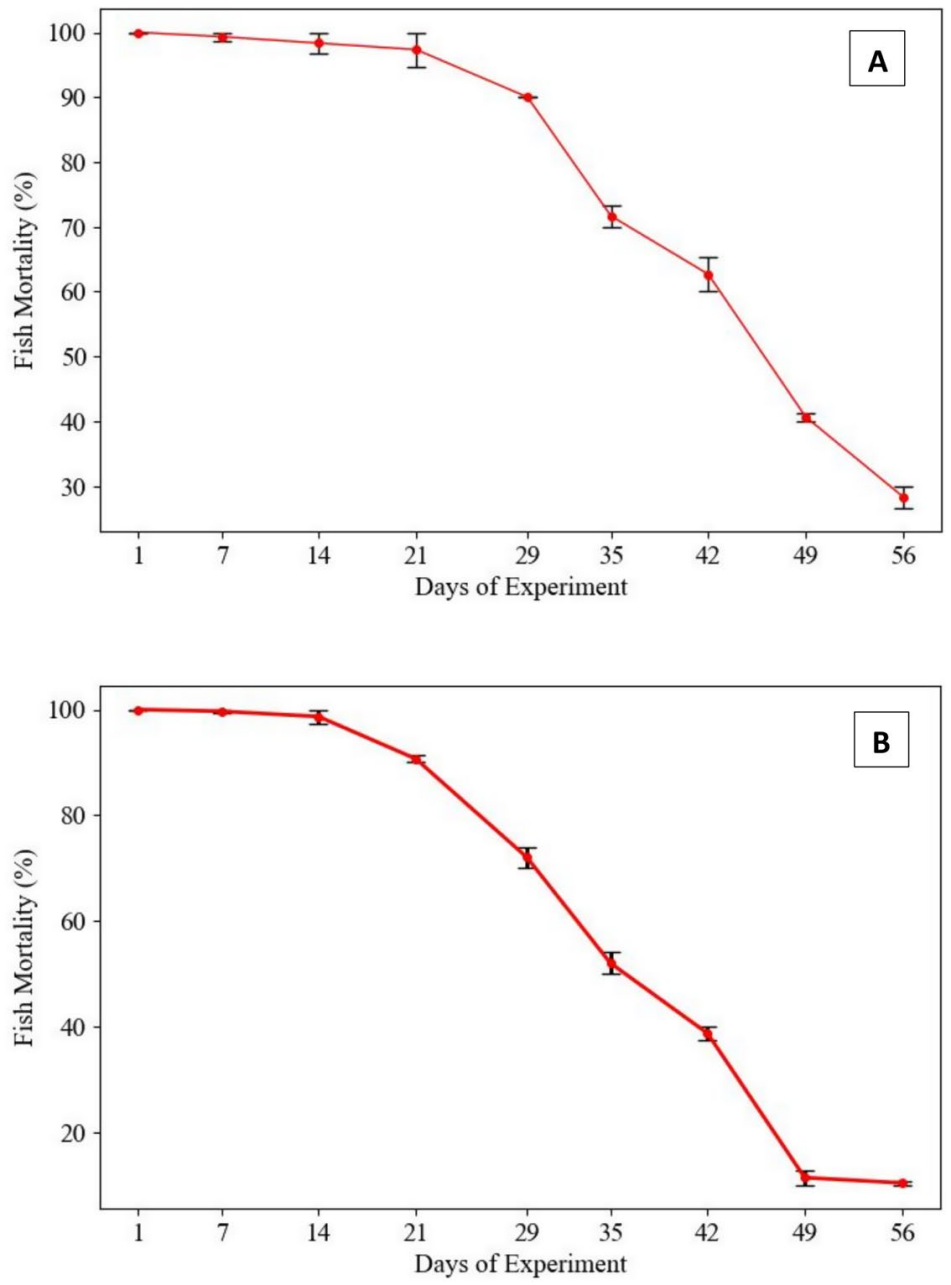
Mortality rates in *Labeo rohita* infected with Gram-negative pathogens following repeated *in-vitro* subculturing. **(A)** *Proteus penneri*: Mortality rates declined over repeated subcultures, with a consistent drop observed after day 29. By day 56, mortality stabilized at 30%, indicating a partial attenuation of virulence in this Gram-negative pathogen. **(B)** *Escherichia coli*: Fish mortality decreased progressively from the initial challenge, yet some virulence was retained, with a final mortality rate of 10% by day 56. This suggests greater resilience in virulence retention among Gram-negative pathogens.

In the fish challenge assay, *L. rohita* were intraperitoneally injected with a suspension of Gram-negative bacterial strains (*Proteus penneri* and *Escherichia coli*) at a standardized concentration of 1.2 × 10^7^ CFU/fish, administered at 7-day intervals. Mortality patterns revealed a progressive decline in virulence over time for both bacterial species. For *P. penneri*, fish mortality gradually decreased from the 29th day post-injection, with a 30% mortality rate observed by the 56th day. In the case of *E. coli*, fish mortality began to decline from the 21st day, with a markedly lower mortality rate of 10% recorded on the 56th day. These findings suggest a potential attenuation of virulence in the tested Gram-negative pathogens following repeated *in-vitro* subculturing, highlighting its impact on pathogen-host interactions and mortality outcomes (Figure 2).

### 3.3 Antibiogram assay of Gram-positive bacteria

The antibiogram profile of *Lactococcus lactis* revealed resistance to several antibiotics, including Rifampicin (RIF5), Colistin (CL10), Polymyxin B (PB300), Trimethoprim (TR5), Nalidixic Acid (NA30), Fosfomycin (FO200), and Doxycycline (DO10). However, the strain exhibited sensitivity to a broad spectrum of antibiotics, including Ampicillin (AMP), Amoxicillin (AMX), Piperacillin (PIT100/10), Ceftazidime (CAZ30), Cefepime (CPM30), Cefixime (CFM5), Streptomycin (S25), Ofloxacin (OF2), Tetracycline (TE), Erythromycin (E10), Chloramphenicol (C30), Ciprofloxacin (CIP5), Netilmicin Sulphate (NET30), Tobramycin (TOB10), Nitrofurantoin (NIT), and Gentamicin (GEN10). Despite repeated *in-vitro* subculturing for up to 56 days, no measurable alterations were observed in the antimicrobial resistance profile of *L. lactis*, indicating stable resistance and sensitivity patterns over extended subculturing periods (Table 3; Supplementary Table 1).

**Table 3:** Comparison of antibiogram assay results between the initial and 56th day of *Lactococcus lactis.*

Antibiogram assay result of isolated *Lactococcus lactis*	Antibiogram assay result of 56th days subcultured *Lactococcus lactis*
*Resistant antibiotic*	*Resistant antibiotic*
Rifampicin (RIF5)	Rifampicin (RIF5)
Fosfomycin (FO200)	Fosfomycin (FO200)
Trimethoprim (TR5)	Trimethoprim (TR5)
Polymixin B (PB300)	Polymixin B (PB300)
Doxycycline (DO10)	Doxycycline (DO10)
Nalidixic acid (NA30)	Nalidixic acid (NA30)
Colistin (CL10)	Colistin (CL10)
*Sensitive antibiotic*	*Sensitive antibiotic*
Ofloxacin (OF2)	Ofloxacin (OF2)
Kanamycin (K30)	Kanamycin (K30)
Ciprofloxacin (CIP5)	Ciprofloxacin (CIP5)
Streptomycin (S25)	Streptomycin (S25)
Ceftazidime (CAZ30)	Ceftazidime (CAZ30)
Tobramycin (TOB10)	Tobramycin (TOB10)
Gentamicin (GEN10)	Gentamicin (GEN10)
Piperacillin (PIT100/10)	Piperacillin (PIT100/10)
Netilmicin sulphate (NET30)	Netilmicin sulphate (NET30)
Nitrofurantoin (NIT200)	Nitrofurantoin (NIT200)
Ampicillin (AMP25)	Ampicillin (AMP25)
Dicloxacillin (D/C)	Dicloxacillin (D/C)
Erythromycin (E10)	Erythromycin (E10)
Amoxycilin (AMC30)	Amoxycilin (AMC30)
Cefixime (CFM5)	Cefixime (CFM5)
Chloramphenicol (C30)	Chloramphenicol (C30)
Tetracycline (TE10)	Tetracycline (TE10)

Antibiogram assay of *L. lactis*		
Antibiotic	Isolated bacteria	After 56th days of subculture
Rifampicin (RIF5)	R	R
Fosfomycin (FO200)	R	R
Trimethoprim (TR5)	R	R
Polymixin B (PB300)	R	R
Doxycycline (DO10)	R	R
Nalidixic acid (NA30)	R	R
Colistin (CL10)	R	R
Ofloxacin (OF2)	S	S
Kanamycin (K30)	S	S
Ciprofloxacin (CIP5)	S	S
Streptomycin (S25)	S	S
Ceftazidime (CAZ30)	S	S
Tobramycin (TOB10)	S	S
Gentamicin (GEN10)	S	S
Piperacillin (PIT100/10)	S	S
Netilmicin sulphate (NET30)	S	S
Nitrofurantoin (NIT200)	S	S
Ampicillin (AMP25)	S	S
Dicloxacillin (D/C)	S	S
Erythromycin (E10)	S	S
Amoxycilin (AMC30)	S	S
Cefixime (CFM5)	S	S
Chloramphenicol (C30)	S	S
Tetracycline (TE10)	S	S

**Figure 3:**
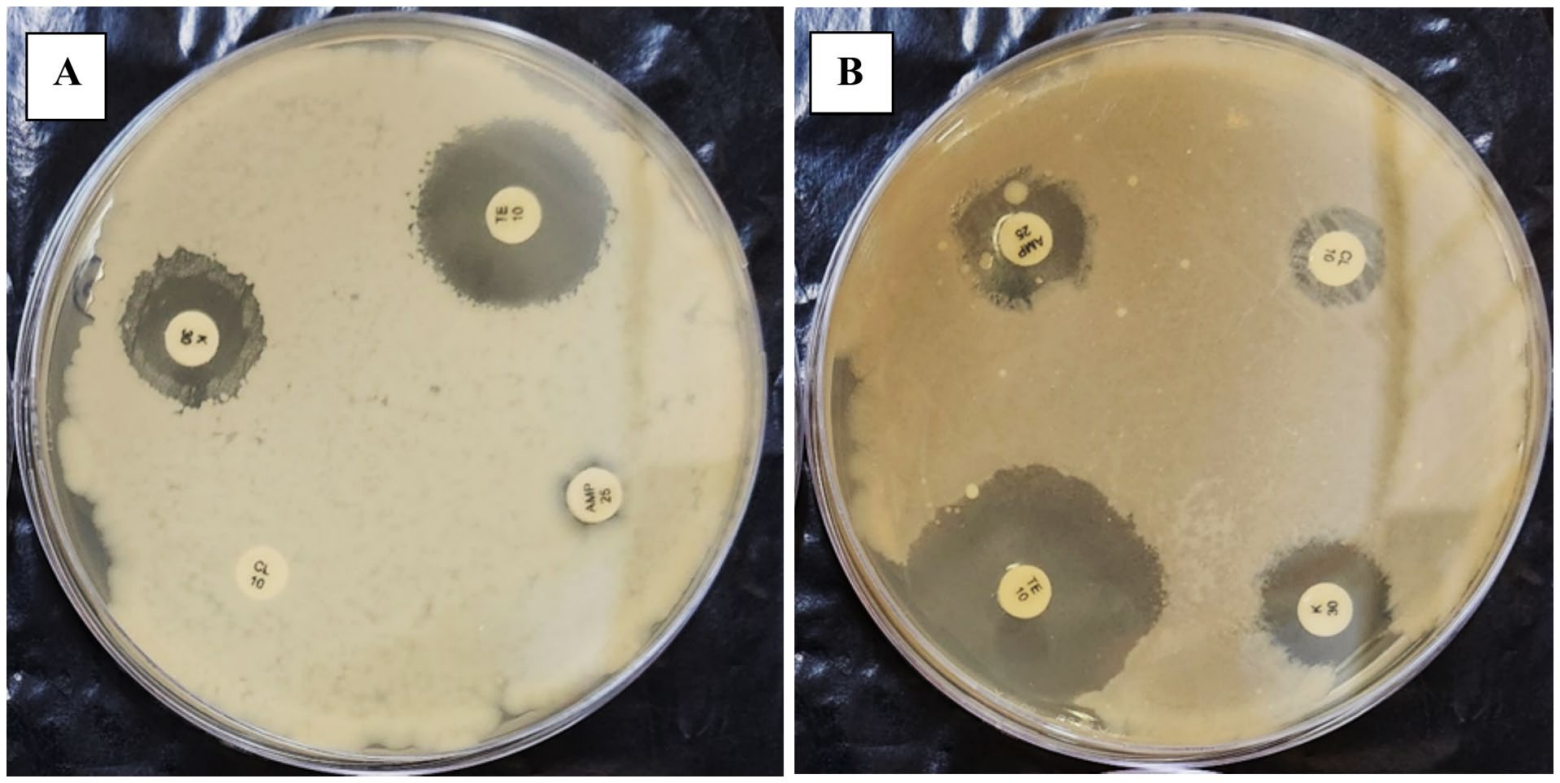
The figure illustrates the effect of repeated *in-vitro* subculture of *Enterococcus gallinarum*. Panels **(A,B)** show that 1st-day *E. gallinarum* show resistance against CL10.

For *Enterococcus gallinarum* isolated *from Hypophthalmichthys nobilis,* the antibiogram assay demonstrated resistance to Ampicillin (AMP25), Dicloxacillin (D/C), Trimethoprim (TR5), Polymyxin B (PB300), Cefixime (CFM5), Tetracycline (TE10), Nalidixic Acid (NA30), and Colistin (CL10), Erythromycin (E10), Doxycycline (DO10). Conversely, it was sensitive to Rifampicin (RIF5), Ofloxacin (OF2), Kanamycin (K30), Ciprofloxacin (CIP5), Streptomycin (S25), Ceftazidime (CAZ30), Tobramycin (TOB10), Gentamicin (GEN10), Fosfomycin (FO200), Piperacillin (PIT100/10), Cefepime (CPM30), Netilmicin Sulphate (NET30), Nitrofurantoin (NIT200), Chloramphenicol (C30), and Amoxicillin (AMC30). Notably, after 42 days of subculturing, the strain demonstrated a significant response to Colistin (CL10), indicating a potential adaptive change in resistance over time (Table 4; Figure 3; Supplementary Table 2).

**Table 4:** Comparison of antibiogram assay results between the initial and 56th-day subculture of isolated *Enterococcus gallinarum.*

Antibiogram assay result of isolated *Enterococcus gallinarum*	Antibiogram assay result of 56^th^ days subcultured *Enterococcus gallinarum*
*Resistant antibiotic*	*Resistant antibiotic*
Ampicilin (AMP25)	Ampicilin (AMP25)
Dicloxacillin (D/C)	Dicloxacillin (D/C)
Trimethoprim (TR5)	Trimethoprim (TR5)
Polymyxin B (PB300)	Erythromycin (E10)
Erythromycin (E10)	Cefixime (CFM5)
Cefixime (CFM5)	Tetracycline (TE10)
Tetracycline (TE10)	
Colistin (CL10)	Polymyxin B (PB300)
Doxycycline (DO10)	Doxycycline (DO10)
Nalidixic acid (NA30)	Nalidixic acid (NA30)
*Sensitive antibiotic*	*Sensitive antibiotic*
Rifampicin (RIF5)	Rifampicin (RIF5)
Ofloxacin (OF2)	Ofloxacin (OF2)
Kanamycin (K30)	Kanamycin (K30)
Ciprofloxacin (CIP5)	Ciprofloxacin (CIP5)
Streptomycin (S25)	Streptomycin (S25)
Ceftazidime (CAZ30)	Ceftazidime (CAZ30)
Tobramycin (TOB10)	Tobramycin (TOB10)
Gentamycin (GEN10)	Gentamycin (GEN10)
Fosfomycin (FO200)	Fosfomycin (FO200)
Piperacillin (PIT100/10)	Piperacillin (PIT100/10)
Cefipime (CPM30)	Cefipime (CPM30)
Netilmicin sulphate (NET30)	Netilmicin sulphate (NET30)
Nitrofurantion (NIT200)	Nitrofurantion (NIT200)
Chloramphenicol (C30)	Chloramphenicol (C30)
Amoxycillin (AMC30)	Amoxycillin (AMC30)
	Colistin (CL10)

Antibiogram assay of *E. gallinarum*		
Antibiotic	Isolated bacteria	After 56th days of subculture
Ampicilin (AMP25)	R	R
Dicloxacillin (D/C)	R	R
Trimethoprim (TR5)	R	R
Polymyxin B (PB300)	R	R
Erythromycin (E10)	R	R
Cefixime (CFM5)	R	R
Tetracycline (TE10)	R	R
Colistin (CL10)	R	S
Doxycycline (DO10)	R	R
Nalidixic acid (NA30)	R	R
Rifampicin (RIF5)	S	S
Ofloxacin (OF2)	S	S
Kanamycin (K30)	S	S
Ciprofloxacin (CIP5)	S	S
Streptomycin (S25)	S	S
Ceftazidime (CAZ30)	S	S
Tobramycin (TOB10)	S	S
Gentamycin (GEN10)	S	S
Fosfomycin (FO200)	S	S
Piperacillin (PIT100/10)	S	S
Cefipime (CPM30)	S	S
Netilmicin sulphate (NET30)	S	S
Nitrofurantion (NIT200)	S	S
Chloramphenicol (C30)	S	S
Amoxycillin (AMC30)	S	S

### 3.4 Antibiogram assay of Gram-negative bacteria

**Figure 4:**
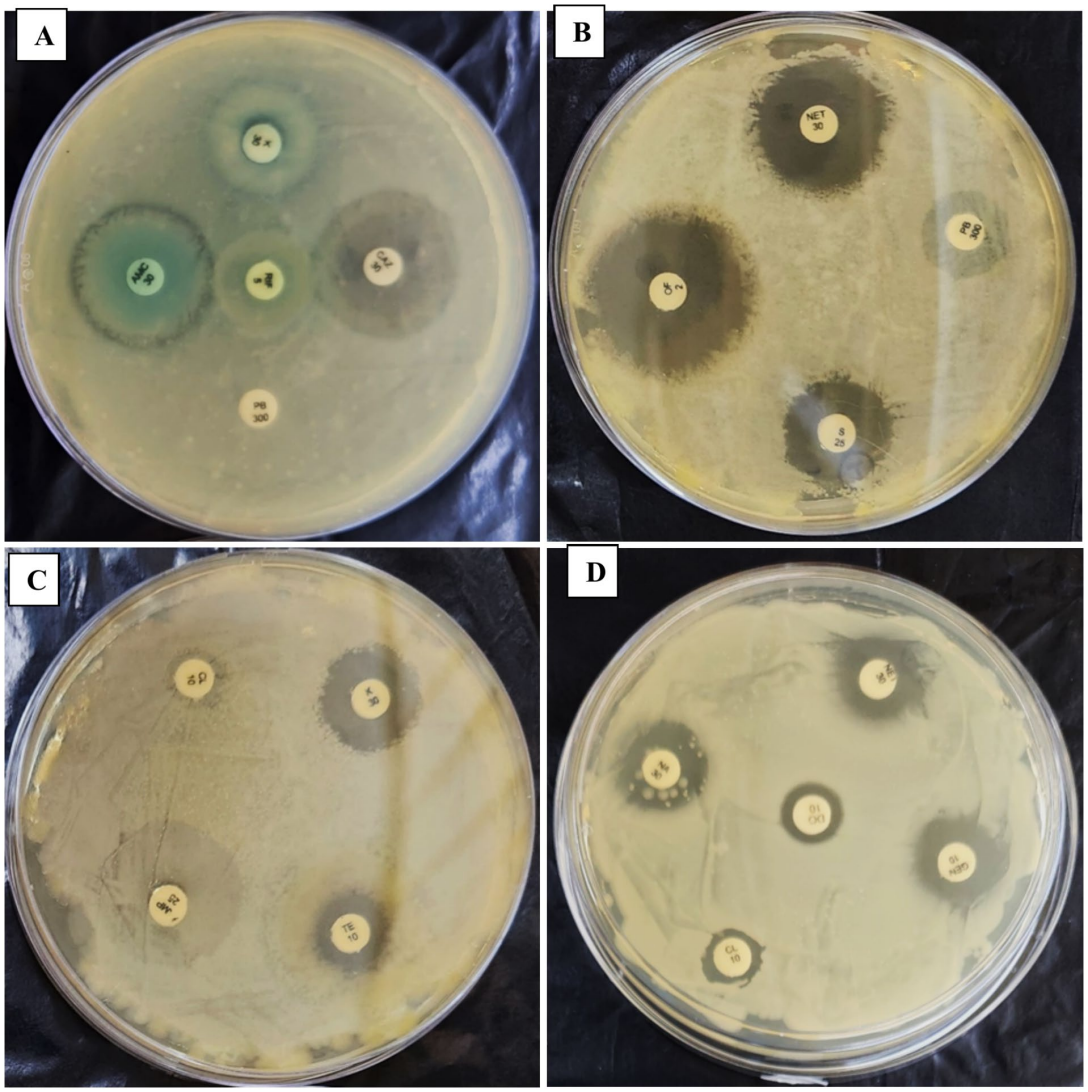
Panels **(A–D)** show the effect of a repeated *in-vitro* subculture of *Proteus penneri* antibiogram resistance profile. 1st day of *P. penneri* show resistance against CL10 & PB300 but after 56th days and sensitive against CL10 & PB300.

The antibiogram assay for *P. penneri*, isolated from *L. catla*, revealed resistance to several antibiotics, including Dicloxacillin (D/C), Ampicillin (AMP25), Fosfomycin (FO200), Polymyxin B (PB300), Trimethoprim (TR5), Tetracycline (TE10), Doxycycline Hydrochloride (DO10), Colistin (CL10), and Erythromycin (E10). The strain displayed intermediate susceptibility to Rifampicin (RIF5), Nitrofurantoin (NIT200), and Amoxicillin (AMC30). However, *Proteus penneri* was sensitive to Kanamycin (K30), Ofloxacin (OF2), Cefixime (CFM5), Gentamicin (GEN10), Tobramycin (TOB10), Nalidixic Acid (NA30), Streptomycin (S25), Chloramphenicol (C30), Ciprofloxacin (CIP5), Piperacillin (PIT100/10), and Ceftazidime (CAZ30). Interpretation of results adhered to the National Committee for Clinical Laboratory Standards (NCCLS). Notably, after 42 days of repeated subculturing, a significant shift in the susceptibility profile of *P. penneri* was observed, with the strain showing susceptibility to Polymyxin B (PB300) and Colistin (CL10), previously categorized as resistant (Table 5; Figure 4; Supplementary Table 3).

**Table 5:** Comparison of antibiogram assay results between initial and 56th-day of isolated *Proteus penneri* subculture.

Antibiogram assay result of isolated *Proteus penneri*	Antibiogram assay result of 64th day subcultured *Proteus penneri*
*Resistant antibiotic*	*Resistant antibiotic*
Dicloxacillin (D/C)	Dicloxacillin (D/C)
Ampicillin (AMP25)	Ampicillin (AMP25)
Fosfomycin (FO200)	Fosfomycin (FO200)
PolymyxinB (PB300)	Trimethoprime (TR5)
Trimethoprime (TR5)	Tetracycline (TE10)
Tetracycline (TE10)	Doxycyclin Hydrochloride (DO10)
Doxycyclin Hydrochloride (DO10)	Erythromycin (E10)
Erythromycin (E10)	*Sensitive antibiotic*
Colistin (CL10)	Cefixime (CFM5)
*Sensitive antibiotic*	Gentamicin (GEN10)
Cefixime (CFM5)	Tobramycin (TOB10)
Gentamicin (GEN10)	Nalidixic acid (NA30),
Tobramycin (TOB10)	Kanamycin (K 30)
Nalidixic acid (NA30),	Streptomycin (S25)
Kanamycin (K 30)	Chloramphenicol (C30)
Streptomycin (S25)	Ciprofloxacin (CIP5)
Chloramphenicol (C30)	Piperacillin (PIT100/10)
Ciprofloxacin (CIP5)	Ceftazidime (CAZ30)
Piperacillin (PIT100/10)	Cefepime (CPM30)
Ceftazidime (CAZ30)	Colistin (CL10)
Cefepime (CPM30)	PolymyxinB (PB300)
Ofloxacin (OF2)	Ofloxacin (OF2)
*Intermediate antibiotic*	*Intermediate antibiotic*
Nitrofurantoin (NIT200)	Nitrofurantoin (NIT200)
Amoxycilin (AMC30)	Amoxycilin (AMC30)
Rifampicin (RIF5)	Rifampicin (RIF5)

Antibiogram assay of *P. penneri*		
Antibiotic	Isolated bacteria	After 56th days of subculture
Dicloxacillin (D/C)	R	R
Ampicillin (AMP25)	R	R
Fosfomycin (FO200)	R	R
PolymyxinB (PB300)	R	S
Trimethoprime (TR5)	R	R
Tetracycline (TE10)	R	R
Doxycyclin Hydrochloride (DO10)	R	R
Erythromycin (E10)	R	R
Colistin (CL10)	R	S
Cefixime (CFM5)	S	S
Gentamicin (GEN10)	S	S
Tobramycin (TOB10)	S	S
Nalidixic acid (NA30),	S	S
Kanamycin (K 30)	S	S
Streptomycin (S25)	S	S
Chloramphenicol (C30)	S	S
Ciprofloxacin (CIP5)	S	S
Piperacillin (PIT100/10)	S	S
Ceftazidime (CAZ30)	S	S
Cefepime (CPM30)	S	S
Ofloxacin (OF2)	S	S
Nitrofurantoin (NIT200)	I	I
Amoxycilin (AMC30)	I	I
Rifampicin (RIF5)	I	I

**Figure 5:**
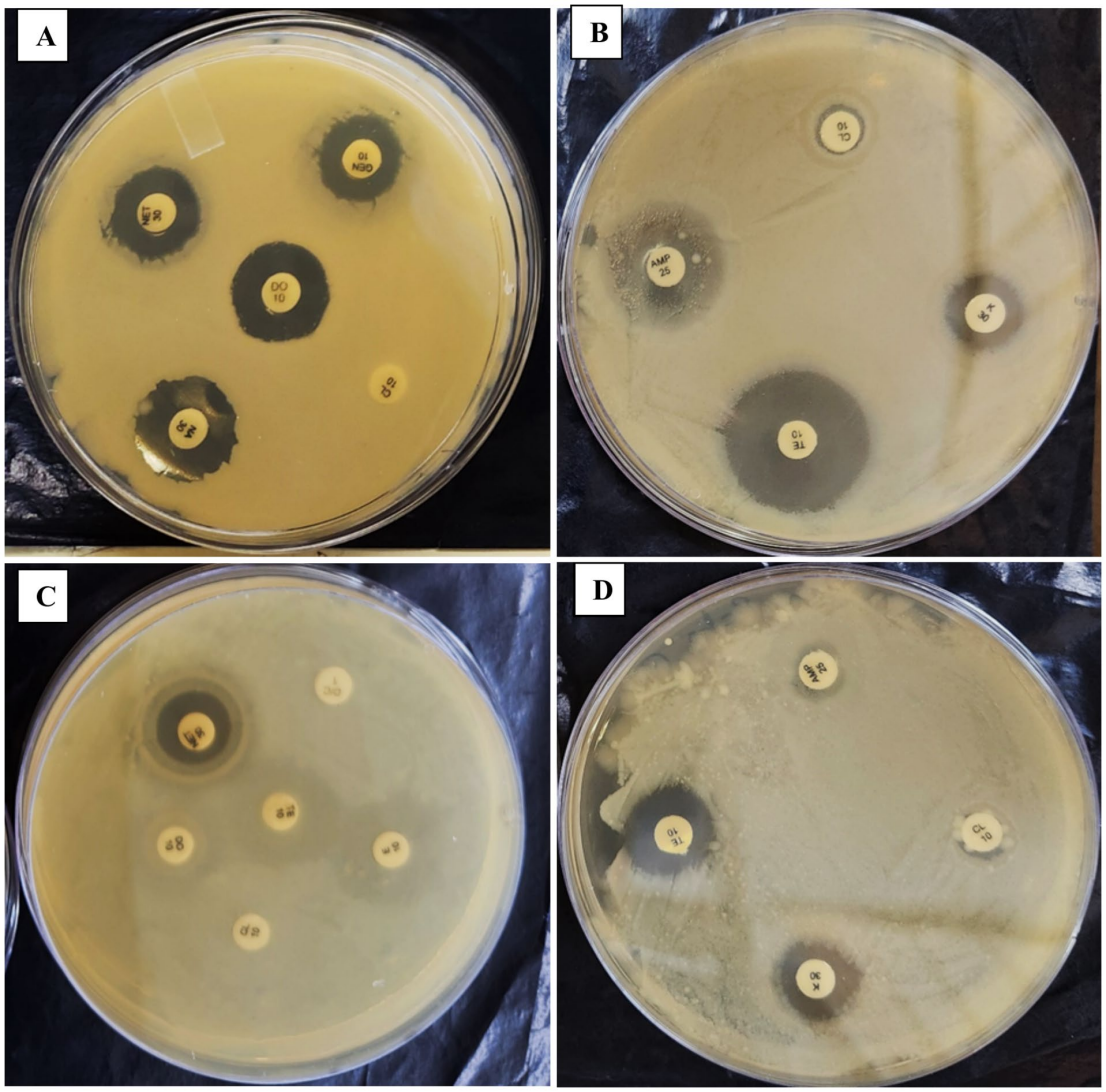
Panels **(A–D)** show the effect of a repeated *in-vitro* subculture of *Escherichia coli* antibiogram resistance profile. 1st day of *E. coli* show intermediate resistance against CL10 & TE10 but after 56th days exhibit sensitive against CL10 & TE10.

For *Escherichia coli*, isolated from *Carassius auratus*, resistance was observed against Rifampicin, Erythromycin (E10), Fosfomycin (FO200), Trimethoprim (TR5), Cefixime (CFM5), Nalidixic Acid (NA30), Doxycycline (DO10), Piperacillin (PIT100/10), and Ampicillin (AMP25). Intermediate susceptibility was recorded for Tetracycline (TE10), Kanamycin (K30), Colistin (CL10), and Cefixime (CFM5). However, the strain was sensitive to Nitrofurantoin (NIT200), Ofloxacin (OF2), Streptomycin (S25), Tobramycin (TOB10), Gentamicin (GEN10), and Netilmicin Sulfate (NET30), Ampicillin (AMP25), Chloramphenicol (C30), Ciprofloxacin (CIP5), Ceftazidime (CAZ30), Amoxycilin (AMC30), PolymyxinB (PB300), Ofloxacin (OF2). After 42 days of *in-vitro* subculturing, *E. coli* demonstrated significant changes in its antimicrobial resistance profile. The strain, which was initially resistant to Tetracycline (TE) and Colistin (CL10), exhibited sensitivity to these antibiotics post-subculturing (Table 6; Figure 5; Supplementary Table 4).

**Table 6:** Comparison of antibiogram assay results between the initial and 56th-day of isolated *Escherichia coli* subculture.

Antibiogram assay result of isolated *E. coli*	Antibiogram assay result of 56th day subcultured *E. coli*
*Resistant antibiotic*	*Resistant antibiotic*
Rifampicin (RIF5)	Rifampicin (RIF5)
Dicloxacillin (D/C)	Dicloxacillin (D/C)
Erythromycin (E10)	Erythromycin (E10)
Fosfomycin (FO200)	Fosfomycin (FO200)
Trimethoprim (TR5)	Trimethoprim (TR5)
Cefixime (CFM5)	Cefixime (CFM5)
Nalidixic acid (NA30)	Nalidixic acid (NA30)
Doxycycline, (DO10)	Doxycycline, (DO10)
Piperacillin (PIT100/10)	Piperacillin (PIT100/10)
Ampicillin (AMP25)	Ampicillin (AMP25)
*Sensitive antibiotic*	*Sensitive antibiotic*
Streptomycin (S25)	Streptomycin (S25)
Tobramycin (TOB10)	Tobramycin (TOB10)
Gentamycin (GEN10)	Gentamycin (GEN10)
Nitrofurantion (NIT200)	Nitrofurantion (NIT200)
Ofloxacin (OF2)	Ofloxacin (OF2)
Chloramphenicol (C30)	Chloramphenicol (C30)
Ciprofloxacin (CIP5)	Ciprofloxacin (CIP5)
Ceftazidime (CAZ30)	Ceftazidime (CAZ30)
Amoxycilin (AMC30)	Amoxycilin (AMC30)
PolymyxinB (PB300)	PolymyxinB (PB300)
*Intermediate antibiotic*	Tetracycline (TE10)
Kanamycin (K30)	Colistin (CL10)
Colistin (CL10)	*Intermediate antibiotic*
Cefixime (CFM5)	Kanamycin (K30)
Tetracycline (TE10)	Cefixime (CFM5)

Antibiogram assay of *Proteus penneri*		
Antibiotic	Isolated bacteria	After 56th days subculture
Rifampicin (RIF5)	R	R
Dicloxacillin (D/C)	R	R
Erythromycin (E10)	R	R
Fosfomycin (FO200)	R	R
Trimethoprim (TR5)	R	R
Cefixime (CFM5)	R	R
Nalidixic acid (NA30)	R	R
Doxycycline, (DO10)	R	R
Piperacillin (PIT100/10)	R	R
Ampicillin (AMP25)	R	R
Streptomycin (S25)	S	S
Tobramycin (TOB10)	S	S
Gentamycin (GEN10)	S	S
Nitrofurantion (NIT200)	S	S
Ofloxacin (OF2)	S	S
Chloramphenicol (C30)	S	S
Ciprofloxacin (CIP5)	S	S
Ceftazidime (CAZ30)	S	S
Amoxycilin (AMC30)	S	S
PolymyxinB (PB300)	S	S
Colistin (CL10)	I	S
Tetracycline (TE10)	I	S
Kanamycin (K30)	I	S
Cefixime (CFM5)	I	S

### 3.5 Antimicrobial gene expression

Quantitative PCR (qPCR) was conducted to assess the expression of antimicrobial resistance genes in both Gram-positive and Gram-negative bacterial strains at the beginning and end of the experiment. The results showed no significant changes in the expression levels of antimicrobial resistance genes after repeated subculturing of the bacterial strains under controlled *in-vitro* conditions. Both Gram-positive and Gram-negative bacteria maintained a consistent expression profile for sulfonamide, tetracycline, quinolone, olaquindox, aminoglycoside, beta-lactam, and chloramphenicol resistance genes throughout the subculture period (Table 2; Supplementary Tables 5–8). This stability in gene expression suggests that repeated subculturing in the absence of selective pressure unlikely causes genetic modifications in the bacteria. However, it is important to note that while no substantial changes were detected in mRNA level, the bacterial phenotypic expression may still be affected. This indicates that repeated subculturing in neutral conditions does not necessarily lead to genetic evolution concerning antimicrobial resistance genes but could potentially influence the phenotype in response to environmental cues or stressors (Braz et al., 2020; Frenkel et al., 2021).

## 4 Discussion

The present study examines the effects of repeated *in-vitro* subculturing on the virulence and antimicrobial resistance profiles of Gram-negative and Gram-positive fish pathogens, specifically *Lactococcus lactis*, *Enterococcus gallinarum*, *Proteus penneri*, and *Escherichia coli*. The findings from this study provide significant insights into the dynamic nature of pathogen-host interactions, the stability of antimicrobial resistance (AMR), and the potential consequences of prolonged laboratory subculturing on bacterial virulence and resistance mechanisms. This discussion section interprets the results in the context of existing research, offering valuable perspectives on bacterial adaptation, AMR, and the implications for aquaculture health management. Repeated *in-vitro* subculturing of fish pathogens can notably influence their virulence and AMR profiles, affecting aquaculture management strategies. Studies have shown the increasing threat of multidrug-resistant pathogens, including *Vibrio* spp.*, Aeromonas* spp., and *Enterobacter cloacae* (Baskaran and Mohan, 2012; Sherif and Kassab, 2023; Mabrok et al., 2024). The potential of probiotics (*Lactobacillus*, *Bacillus* spp.) and natural antimicrobial agents, such as algal and plant extracts, to reduce AMR has been emphasized (Vine et al., 2006; El-Sapagh et al., 2023). Understanding the genetic and phenotypic adaptations during subculturing is essential for developing effective AMR monitoring and pathogen control strategies.

In this study, we observed a gradual decline in mortality rates among *Labeo rohita* challenged with *L. lactis* and *E. gallinarum*, two Gram-positive pathogens, over successive *in-vitro* subcultures. Initially, both pathogens induced 100% mortality within the first 2 weeks of infection. However, as the fish challenge repeated with the same bacterial strains at 7-day intervals, a decline in mortality was noted, with 0% mortality recorded by day 56 (Figure 1). This reduction in virulence suggests a potential attenuation of pathogenicity in response to prolonged subculture in neutral conditions, consistent with similar studies demonstrating the attenuation of virulence factors in bacterial strains after multiple *in-vitro* passages (Sung et al., 2002; Ósrstavik and Ósrstavik, 1982). The loss of virulence is often attributed to the decreased expression of critical virulence factors, such as adhesins, toxins, and surface proteins, which may occur when bacteria are cultured under non-selective, stress-free conditions (Westergren and Svanberg, 1983). Similarly, our study on Gram-negative pathogens, specifically *P. penneri* and *E. coli*, revealed a progressive decline in virulence, albeit not to the extent observed with Gram-positive bacteria. Mortality in fish challenged with *P. penneri* and *E. coli* gradually decreased, with the latter reaching a mortality rate of 10% by day 56 (Figure 2). Although this represents a significant reduction in virulence compared to initial infections, *P. penneri* and *E. coli* maintained a more robust pathogenic profile than their Gram-positive counterparts. These findings suggest that Gram-negative pathogens may retain certain virulence traits even after multiple subcultures, which may be due to the inherent stability of their virulence-associated genes or mechanisms (Koga et al., 1989; Yamashita et al., 1996). Moreover, Gram-negative bacteria possess additional mechanisms, such as efflux pumps and outer membrane protection, which might provide greater resilience to environmental stressors, including *in-vitro* subculturing (McLean et al., 2019). The variation in the attenuation of virulence between Gram-positive and Gram-negative bacteria observed in this study is consistent with previous research highlighting the differential effects of *in-vitro* subculturing on pathogenicity (Sung et al., 2002; Frenkel et al., 2021). This difference could be linked to the distinct cellular structures and pathogenic mechanisms of these two bacterial groups, with Gram-negative bacteria often exhibiting more complex virulence strategies that are less susceptible to attenuation under laboratory conditions (Frenkel et al., 2021).

The antibiogram assays conducted in this study revealed significant insights into the stability and variability of antimicrobial resistance profiles in the tested fish pathogens. *L. lactis* exhibited a consistent resistance pattern throughout all subculturing periods, demonstrating resistance to Rifampicin, Colistin, Polymyxin B, Trimethoprim, Nalidixic Acid, Fosfomycin, and Doxycycline. Remarkably, the strain-maintained stability in resistance to these antibiotics even after prolonged subculturing for up to 56 days. This suggests a robust, inherent resistance mechanism that is not influenced by the absence of selective pressure (Table 3). This finding aligns with previous reports indicating the stability of resistance traits in bacteria under laboratory conditions, particularly when plasmid-mediated mechanisms or chromosomal mutations confer resistance (Braz et al., 2020). In contrast, *E. gallinarum* demonstrated a more dynamic response to repeated subculturing. Initially, the strain was resistant to several antibiotics, including Ampicillin, Dicloxacillin, Polymyxin B, Tetracycline, and Nalidixic Acid. However, after 42 days of subculturing, the strain exhibited a significant response to Colistin, indicating a potential adaptive change in its resistance profile over time (Table 4; Figure 3). This implies that *E. gallinarum* may be able to adjust its resistance profile in response to environmental shifts or stressors, albeit more subtly than *L. lactis*. The emergence of significant resistance to Colistin in *E. gallinarum* resonates with findings from other studies that emphasize the role of adaptive resistance mechanisms in response to prolonged subculturing or environmental changes (Braz et al., 2020). The resistance patterns were similarly stable during subculturing for the Gram-negative pathogens *P. penneri* and *E. coli*. *P. penneri* resisted Dicloxacillin, Ampicillin, Fosfomycin, and Polymyxin B, showing significant susceptibility to Rifampicin and Nitrofurantoin. Notably, after 42 days of subculturing, there was a shift in the resistance profile, with the strain demonstrating significant susceptibility to Polymyxin B and Colistin, both previously deemed resistant (Table 5; Figure 4). These changes suggest that *P. penneri* may adjust to laboratory conditions, although the alterations were not as pronounced as those observed in *E. gallinarum*. Similarly, *E. coli* exhibited antibiotic resistance, including Rifampicin, Erythromycin, Fosfomycin, and Trimethoprim, with significant susceptibility to Colistin and Tetracycline. After 42 days of subculturing, a notable shift in the antimicrobial profile was detected, with the strain showing sensitivity to Tetracycline and Colistin, which had previously been significant (Table 6; Figure 5). This change in resistance, particularly the emergence of susceptibility to antibiotics after prolonged subculturing, suggests that extended exposure to non-selective conditions could potentially reverse some resistance traits, especially those linked to efflux pumps or surface modifications (Wickham, 2021; Hill, 2017). These findings underscore the plasticity of Gram-negative bacterial resistance mechanisms, which may evolve in response to prolonged subculturing without direct selective pressure.

Bacteria adapt to environmental changes through feedback regulation and phenotypic variation (Smits et al., 2006; López-Maury et al., 2008). Prolonged subculturing can drive mutations and epigenetic changes, enhancing resistance or reducing virulence (Roberti et al., 2006; Knopp and Andersson, 2018). Under selective pressure, these shifts pose challenges for pathogen management in aquaculture, where AMR persistence is critical (Christaki et al., 2020). Understanding these dynamics helps inform strategies to minimize pathogen evolution and maintain effective treatments (Hughes and Andersson, 2017).

No discernible changes in the expression levels of resistance genes of both Gram-positive and Gram-negative bacteria (*sul1, sul2, tetM, tetW, qepA, qnrS, oqxA, oqxB, aac, ami, blaP*) were found by the RT-qPCR data over the 56-day subculturing period. Given this stability, it is likely that the transcriptional activity of resistance genes is generally unaffected by external selective pressure, such as exposure to antibiotics (Table 2). This stability in gene expression suggests that the observed changes in antimicrobial resistance profiles did not arise from genetic alterations in resistance genes. Instead, these changes may reflect phenotypic adaptations in response to environmental conditions. This finding aligns with previous studies that have indicated phenotypic changes, such as altered resistance profiles, can occur without corresponding genetic mutations, particularly in the absence of direct selective pressure (Braz et al., 2020; Hughes and Andersson, 2017; Roberti et al., 2006; Knopp and Andersson, 2018). The absence of significant genetic changes in antimicrobial resistance genes after repeated subculturing may imply that resistance mechanisms in these pathogens are primarily stable and do not necessitate frequent genetic adaptation to persist in laboratory conditions. Nonetheless, as highlighted in studies by Hughes and Andersson (2017) and López-Maury et al. (2008), the phenotypic expression of resistance can still be influenced by factors such as environmental stressors, nutrient availability, and the presence of specific bacterial signaling molecules, even in the absence of genetic evolution.

The findings of this study have significant implications for pathogen management in aquaculture. First, the reduction of virulence observed in *L. lactis* and *E. gallinarum* under repeated subculturing indicates that ongoing monitoring of virulence factors in aquaculture pathogens is crucial, as pathogens may become less virulent in laboratory conditions, potentially resulting in inaccurate evaluations of their pathogenicity in natural environments. Second, the evolving nature of antimicrobial resistance, especially in Gram-negative pathogens like *P. penneri* and *E. coli*, highlights the need for close surveillance of resistance patterns over time. Prolonged subculturing, even without selective pressure, can cause shifts in resistance profiles, which may significantly affect treatment strategies in aquaculture. Finally, the persistence of antimicrobial resistance genes despite phenotypic changes in resistance underscores the complexity of bacterial adaptation.

The study demonstrates how important environmental context is in determining how microorganisms behave. Ongoing exposure to antibiotics in aquaculture settings may encourage the production and survival of resistance genes. On the other hand, bacteria could return to a less virulent and resistant condition under carefully monitored lab settings free from human immunological pressure and drugs. The findings have a big impact on aquaculture as well as human disease control methods. Developing attenuated live vaccines for fish may be made easier with an understanding of how subculturing reduces virulence. For example, serially passaged bacteria that are still immunogenic but no longer pathogenic might be good candidates for vaccine development (Ellis, 2001). The differences in antibiotic susceptibility provide further information for antimicrobial management. By determining the circumstances under which resistance decays, we can improve antibiotic rotation or withdrawal tactics to lessen the pressure of resistance selection in aquaculture settings.

It is necessary to recognize a number of limitations despite the study’s extensive breadth. The results may not be as generalisable to other aquaculture systems due to the fact that just one host species (*L. rohita*) was used. Several fish species with different immunological capacities should be included in future research to gain a deeper understanding of host-pathogen interactions. Although not investigated in this work, quorum sensing or epigenetic pathways may also have an impact on phenotypic changes in bacterial virulence and resistance. Under laboratory circumstances, examining these processes may provide insight into reversible and non-genetic adaptations in bacteria. Additionally, while qPCR provided valuable insights into gene expression, whole-genome sequencing or transcriptomic approaches would offer a more holistic view of the genetic changes accompanying prolonged subculturing. These methods could reveal mutations, mobile genetic elements, or regulatory pathway alterations that may not be evident through targeted qPCR alone. Lastly, Real aquaculture environments have extra complications such as biofilm development, microbial competition, and host immunological interactions, even though the study mimicked *in-vitro* subculturing. Future experimental designs that take these factors into account will have greater ecological relevance and translational usefulness. It stresses the need for further research into the molecular mechanisms driving resistance and the potential influence of environmental factors on resistance phenotypes. As antimicrobial resistance remains a global challenge, understanding the elements affecting bacterial resistance and virulence in aquaculture systems is vital for developing more effective management strategies.

This study highlights how repeated *in-vitro* subculturing influences the virulence and antimicrobial resistance of both Gram-positive and Gram-negative fish pathogens. Notably, while Gram-positive pathogens exhibited a more pronounced decrease in virulence, Gram-negative bacteria maintained their pathogenic abilities even after extended subculturing. Although the antimicrobial resistance profiles of both pathogen groups remained largely stable, minor changes were observed, particularly in *E. gallinarum*, *P. penneri* and *E. coli*. These findings emphasize the critical need for continuous monitoring of pathogen virulence and resistance characteristics to ensure effective disease management in aquaculture. Moreover, additional research is necessary to explore the mechanisms underlying these findings and their potential implications for pathogen control in natural ecosystems.

## 5 Conclusion

This study examines how repeated in-vitro subculturing affects the virulence and antimicrobial resistance profiles of Gram-positive and Gram-negative fish pathogens, specifically *L. lactis*, *E. gallinarum*, *P. penneri*, and *E. coli*. This work emphasizes the complex interplay of host-pathogen dynamics, environmental factors, and bacterial adaptability. The findings reveal a clear trend of reduced virulence, particularly among Gram-positive pathogens. *L. lactis* and *E. gallinarum* demonstrated a significant decrease in virulence with successive subcultures, with *L. lactis* virulence diminishing to zero by day 56. This loss of pathogenicity is likely linked to the depletion of essential virulence factors under non-selective conditions. Conversely, Gram-negative pathogens, such as *P. penneri* and *E. coli*, maintained greater virulence, experiencing only slight declines in mortality rates during the same period. This indicates that Gram-negative bacteria are resilient due to their more complex virulence mechanisms. The antimicrobial resistance patterns remained mostly stable across Gram-positive and Gram-negative bacteria, accompanied by slight changes, especially in *P. penneri, E. coli, and E. gallinarum*. Surprisingly, after 56 days of subculturing, *E. gallinarum* exhibits sensitivity to colistin (CL10). Polymyxin B (PB300) and colistin (CL10) are both sensitive to *Proteus pinneri*. *Escherichia coli* returned to being susceptible to tetracycline (TE10) and colistin (CL10), among other antibiotics. This finding shows that, even in the absence of genetic alterations, environmental influences during subculturing may affect resistance characteristics. These results illustrate the dynamic relationship between bacterial adaptation, virulence, and antimicrobial resistance, emphasizing the need for continuous monitoring of pathogen virulence and resistance in aquaculture to develop effective management strategies against the growing challenge of antimicrobial resistance. We can create sustainable aquaculture methods, optimize the use of antibiotics, and create more effective disease control tactics by knowing how bacteria adapt outside of their natural habitats.

## Data Availability

All data is contained within the manuscript and additional files. The nucleotide sequences of the 16S rDNA gene and accession numbers of bacterial isolates have been deposited in the GenBank database under accession numbers DR999571, OR999570, OP554277, and OR794370.
