# Supplementary material for: Impact of repeated in-vitro bacterial culture on virulence and antibiotic resistance characteristics: a study of Gram-positive and Gram-negative fish pathogens
Source: Front Microbiol. 2025 May 21;16:1601681. doi: 10.3389/fmicb.2025.1601681 (PMC12133814; doi:10.3389/fmicb.2025.1601681)
Supplement: Supplementary file 1 [file Table_1.docx]

***In vitro* Antibiogram assay of Bacteria**

**Supplementary Table 1**. Antibiogram Assay of *Lactococcus lactis*

| **Antibiogram Assay of *Lactococcus lactis*** | | | | | | | | | |
| --- | --- | --- | --- | --- | --- | --- | --- | --- | --- |
| Name of antibiotic | 0 day | 7^th^ Day | 14^th^ Day | 21^th^ Day | 28^th^ Day | 35^th^ Day | 42th Day | 49^th^ Day | 56^th^ Day |
| Rifampicin (RIF5) | R | R | R | R | R | R | R | R | R |
| Fosfomycin (FO200) | R | R | R | R | R | R | R | R | R |
| Trimethoprim(TR5) | R | R | R | R | R | R | R | R | R |
| Polymixin B (PB300) | R | R | R | R | R | R | R | R | R |
| Doxycycline (DO10) | R | R | R | R | R | R | R | R | R |
| Nalidixic acid (NA30) | R | R | R | R | R | R | R | R | R |
| Colistin (CL10) | R | R | R | R | R | R | R | R | R |
| Ofloxacin (OF2) | S | S | S | S | S | S | S | S | S |
| Kanamycin (K30) | S | S | S | S | S | S | S | S | S |
| Ciprofloxacin (CIP5) | S | S | S | S | S | S | S | S | S |
| Streptomycin (S25) | S | S | S | S | S | S | S | S | S |
| Ceftazidime (CAZ30) | S | S | S | S | S | S | S | S | S |
| Tobramycin (TOB10) | S | S | S | S | S | S | S | S | S |
| Gentamicin (GEN10) | S | S | S | S | S | S | S | S | S |
| Piperacillin (PIT100/10) | S | S | S | S | S | S | S | S | S |
| Netilmicin sulphate (NET30) | S | S | S | S | S | S | S | S | S |
| Nitrofurantoin (NIT200) | S | S | S | S | S | S | S | S | S |
| Ampicillin (AMP25) | S | S | S | S | S | S | S | S | S |
| Dicloxacillin (D/C) | S | S | S | S | S | S | S | S | S |
| Erythromycin (E10) | S | S | S | S | S | S | S | S | S |
| Amoxycilin (AMC30) | S | S | S | S | S | S | S | S | S |
| Cefixime (CFM5) | S | S | S | S | S | S | S | S | S |
| Chloramphenicol (C30) | S | S | S | S | S | S | S | S | S |
| Tetracycline (TE10) | S | S | S | S | S | S | S | S | S |

**Supplementary Table 2**. Antibiogram Assay of *Enterococcus gallinarum*

| **Antibiogram assay of *Enterococcus gallinarum*** | | | | | | | | | |
| --- | --- | --- | --- | --- | --- | --- | --- | --- | --- |
| Name of antibiotic | 1^st^ | 7th | 14th | 21th | 29th | 35th | 42th | 49th | 56th |
| Ampicilin(AMP25) | R | R | R | R | R | R | R | R | R |
| Dicloxacillin (D/C) | R | R | R | R | R | R | R | R | R |
| Trimethoprim (TR5) | R | R | R | R | R | R | R | R | R |
| Polymyxin B (PB300) | R | R | R | R | R | R | R | R | R |
| Erythromycin (E10) | R | R | R | R | R | R | R | R | R |
| Cefixime (CFM5) | R | R | R | R | R | R | R | R | R |
| Tetracycline (TE10) | R | R | R | R | R | R | R | R | R |
| Colistin (CL10) | R | R | R | R | R | R | S | S | S |
| Nalidixic acid (NA30) | R | R | R | R | R | R | R | R | R |
| Doxycycline (DO10) | R | R | R | R | R | R | R | R | R |
| Rifampicin (RIF5) | S | S | S | S | S | S | S | S | S |
| Ofloxacin (OF2) | S | S | S | S | S | S | S | S | S |
| Kanamycin (K30) | S | S | S | S | S | S | S | S | S |
| Ciprofloxacin (CIP5) | S | S | S | S | S | S | S | S | S |
| Streptomycin (S25) | S | S | S | S | S | S | S | S | S |
| Ceftazidime (CAZ30) | S | S | S | S | S | S | S | S | S |
| Tobramycin (TOB10) | S | S | S | S | S | S | S | S | S |
| Gentamycin (GEN10) | S | S | S | S | S | S | S | S | S |
| Fosfomycin (FO200) | S | S | S | S | S | S | S | S | S |
| Piperacillin (PIT100/10) | S | S | S | S | S | S | S | S | S |
| Cefipime (CPM30) | S | S | S | S | S | S | S | S | S |
| Netilmicin sulphate (NET30) | S | S | S | S | S | S | S | S | S |
| Nitrofurantion(NIT200) | S | S | S | S | S | S | S | S | S |
| Chloramphenicol (C30) | S | S | S | S | S | S | S | S | S |
| Amoxycillin (AMC30) | S | S | S | S | S | S | S | S | S |

**Supplementary Table 3**. Antibiogram Assay of *Proteus penneri*

| **Antibiogram Assay of *Proteus penneri*** | | | | | | | | | |
| --- | --- | --- | --- | --- | --- | --- | --- | --- | --- |
| Name of Antibiotics | 1st | 7th | 14th | 21th | 29th | 35th | 42th | 49th | 56th |
| Dicloxacillin (D/C) | R | R | R | R | R | R | R | R | R |
| Ampicillin (AMP25) | R | R | R | R | R | R | R | R | R |
| Fosfomycin (FO200) | R | R | R | R | R | R | R | R | R |
| PolymyxinB (PB300) | R | R | R | R | R | R | S | S | S |
| Trimethoprime (TR5) | R | R | R | R | R | R | R | R | R |
| Tetracycline (TE10) | R | R | R | R | R | R | R | R | R |
| Doxycyclin Hydrochloride (DO10) | R | R | R | R | R | R | R | R | R |
| Erythromycin (E10) | R | R | R | R | R | R | R | R | R |
| Colistin (CL10) | R | R | R | R | R | R | S | S | S |
| Cefixime (CFM5) | R | R | R | R | R | R | R | R | R |
| Gentamicin (GEN10) | S | S | S | S | S | S | S | S | S |
| Tobramycin (TOB10) | S | S | S | S | S | S | S | S | S |
| Nalidixic acid (NA30), | S | S | S | S | S | S | S | S | S |
| Kanamycin (K 30) | S | S | S | S | S | S | S | S | S |
| Streptomycin (S25) | S | S | S | S | S | S | S | S | S |
| Chloramphenicol (C30) | S | S | S | S | S | S | S | S | S |
| Ciprofloxacin (CIP5) | S | S | S | S | S | S | S | S | S |
| Piperacillin (PIT100/10) | S | S | S | S | S | S | S | S | S |
| Ceftazidime (CAZ30) | S | S | S | S | S | S | S | S | S |
| Cefepime (CPM30) | S | S | S | S | S | S | S | S | S |
| Ofloxacin (OF2) | S | S | S | S | S | S | S | S | S |
| Nitrofurantoin (NIT200) | I | I | I | I | I | I | I | I | I |
| Amoxycilin(AMC30) | I | I | I | I | I | I | I | I | I |
| Rifampicin (RIF5) | I | I | I | I | I | I | I | I | I |

**Supplementary Table 4**. Antibiogram Assay of *Escherichia coli*

| **Antibiogram Assay of *E. coli*** | | | | | | | | | |
| --- | --- | --- | --- | --- | --- | --- | --- | --- | --- |
| Name of Antibiotic | 1st | 7th | 14th | 21th | 29th | 35th | 42th | 49th | 56th |
| Rifampicin (RIF5) | R | R | R | R | R | R | R | R | R |
| Dicloxacillin (D/C) | R | R | R | R | R | R | R | R | R |
| Erythromycin (E10) | R | R | R | R | R | R | R | R | R |
| Fosfomycin (FO200) | R | R | R | R | R | R | R | R | R |
| Trimethoprim (TR5) | R | R | R | R | R | R | R | R | R |
| Cefixime (CFM5) | R | R | R | R | R | R | R | R | R |
| Nalidixic acid (NA30) | R | R | R | R | R | R | R | R | R |
| Doxycycline, (DO10) | R | R | R | R | R | R | R | R | R |
| Piperacillin (PIT100/10) | R | R | R | R | R | R | R | R | R |
| Streptomycin (S25) | S | S | S | S | S | S | S | S | S |
| Tobramycin (TOB10) | S | S | S | S | S | S | S | S | S |
| Gentamycin (GEN10) | S | S | S | S | S | S | S | S | S |
| Streptomycin (S25) | S | S | S | S | S | S | S | S | S |
| Nitrofurantion (NIT200) | S | S | S | S | S | S | S | S | S |
| Kanamycin(K30) | I | I | I | I | I | I | I | I | I |
| Colistin (CL10) | I | I | I | I | I | I | S | S | S |
| Cefixime (CFM5) | I | I | I | I | I | I | I | I | I |
| Tetracycline (TE10) | I | I | I | I | I | S | S | S | S |
| Ampicillin (AMP25) | R | R | R | R | R | R | R | R | R |
| Chloramphenicol (C30) | S | S | S | S | S | S | S | S | S |
| Ciprofloxacin (CIP5) | S | S | S | S | S | S | S | S | S |
| Ceftazidime (CAZ30) | S | S | S | S | S | S | S | S | S |
| Amoxycilin(AMC30) | S | S | S | S | S | S | S | S | S |
| PolymyxinB (PB300) | S | S | S | S | S | S | S | S | S |
| Ofloxacin (OF2) | S | S | S | S | S | S | S | S | S |

**Antimicrobial-resistant gene expression study of Bacteria**

**Supplementary Table 5**. Every 7^th^ day interval antimicrobial gene expression result of *Lactococcus lactis*

| **GENE** | 1^st^ Day | 7^th^ Day | 14^th^ Day | 21th Day | 28^th^ Day | 35^th^ Day | 42th Day | 49^th^ Day | 56^th^ Day |
| --- | --- | --- | --- | --- | --- | --- | --- | --- | --- |
| *sul1*  (Sulfonamide) | 1.01 | 1.05 | 1.00 | 1.01 | 1.00 | 1.00 | 1.01 | 1.00 | 1.03 |
| *sul2*  (Sulfonamide) | 1.01 | 1.01 | 1.00 | 1.01 | 1.17 | 1.00 | 1.00 | 1.00 | 1.01 |
| *tetM*  (Tetracycline) | 1.00 | 1.01 | 1.00 | 1.17 | 1.01 | 1.00 | 1.00 | 1.01 | 1.03 |
| *tetW*  (Tetracycline) | 1.03 | 1.01 | 1.00 | 1.08 | 1.01 | 1.01 | 1.00 | 1.08 | 1.16 |
| *qepA*  (Quinolone) | 1.00 | 1.01 | 1.00 | 1.01 | 1.12 | 1.01 | 1.00 | 1.01 | 1.00 |
| *qnrS*  (Quinolone) | 1.00 | 1.02 | 1.18 | 1.02 | 1.01 | 1.09 | 1.00 | 1.01 | 1.03 |
| *oqxA*  (Olaquindox) | 1.00 | 1.07 | 1.00 | 1.14 | 1.12 | 1.24 | 1.00 | 1.01 | 1.05 |
| *oqxB*  (Olaquindox) | 1.00 | 1.00 | 1.00 | 1.00 | 1.17 | 1.15 | 1.71 | 1.20 | 1.05 |
| *aac(6′)-Ib*  (Aminoglycoside) | 1.01 | 1.01 | 1.00 | 1.00 | 1.19 | 1.03 | 1.00 | 1.01 | 1.02 |
| *ami*  (Aminoglycoside) | 1.00 | 1.11 | 1.50 | 1.36 | 1.16 | 1.00 | 1.00 | 1.02 | 1.02 |
| *blaP*  (Beta-lactam) | 1.00 | 1.01 | 1.00 | 1.12 | 1.16 | 0.01 | 1.00 | 1.05 | 1.03 |
| *Cmr*  (Chloramphenicol) | 1.00 | 1.01 | 1.01 | 1.04 | 1.17 | 1.09 | 1.00 | 1.04 | 1.03 |

**Supplementary Table 6**. Every 7^th^ day interval antimicrobial gene expression result of *Enterococcus gallinarum*

| **GENE** | 1^st^ Day | 7^th^  Day | 14^th^ Day | 21th Day | 28^th^ Day | 35^th^ Day | 42th Day | 49^th^ Day | 56^th^ Day |
| --- | --- | --- | --- | --- | --- | --- | --- | --- | --- |
| *sul1*  (Sulfonamide) | 1.003 | 1.023 | 1.002 | 1.084 | 1.226 | 1.082 | 1.003 | 1.107 | 1.037 |
| *sul2*  (Sulfonamide) | 1.002 | 1.089 | 1.003 | 1.077 | 1.129 | 1.112 | 1.004 | 1.033 | 1.039 |
| *tetM*  (Tetracycline) | 1.000 | 1.040 | 1.097 | 1.002 | 1.158 | 1.017 | 1.000 | 1.014 | 1.037 |
| *tetW*  (Tetracycline) | 1.000 | 1.038 | 1.028 | 1.162 | 1.379 | 1.047 | 1.001 | 1.011 | 1.053 |
| *qepA*  (Quinolone) | 1.001 | 1.019 | 1.007 | 1.059 | 1.140 | 1.003 | 1.006 | 1.012 | 1.026 |
| *qnrS*  (Quinolone) | 1.001 | 1.028 | 1.001 | 1.301 | 1.038 | 1.000 | 1.052 | 1.048 | 1.021 |
| *oqxA*  (Olaquindox) | 1.002 | 1.082 | 1.003 | 1.009 | 1.097 | 1.167 | 1.002 | 1.019 | 1.039 |
| *oqxB*  (Olaquindox) | 1.001 | 1.074 | 1.311 | 1.401 | 1.121 | 1.050 | 1.001 | 1.007 | 1.033 |
| *aac(6′)-Ib*  (Aminoglycoside) | 1.000 | 1.044 | 1.002 | 1.195 | 1.009 | 1.000 | 1.067 | 1.001 | 1.034 |
| *ami*  (Aminoglycoside) | 1.006 | 1.154 | 1.001 | 1.164 | 1.120 | 1.055 | 1.003 | 1.082 | 1.021 |
| *blaP*  (Beta-lactam) | 1.000 | 1.004 | 1.001 | 1.043 | 1.142 | 1.050 | 1.002 | 1.061 | 1.025 |
| *Cmr*  (Chloramphenicol) | 1.064 | 1.019 | 1.001 | 1.085 | 1.167 | 1.001 | 1.002 | 1.075 | 1.020 |

**Supplementary Table 7**. Every 7^th^ day interval antimicrobial gene expression result of *Proteus pinneri*

| **GENE** | 1^st^ Day | 7^th^ Day | 14^th^ Day | 21th Day | 28^th^ Day | 35^th^ Day | 42th Day | 49^th^ Day | 56^th^ Day |
| --- | --- | --- | --- | --- | --- | --- | --- | --- | --- |
| *sul1*  (Sulfonamide) | 1.001 | 1.015 | 1.030 | 1.125 | 1.044 | 1.006 | 1.004 | 1.001 | 1.003 |
| *sul2*  (Sulfonamide) | 1.002 | 1.062 | 1.000 | 1.002 | 1.000 | 1.009 | 1.001 | 1.030 | 1.003 |
| *tetM*  (Tetracycline) | 1.000 | 1.052 | 1.006 | 1.097 | 1.000 | 1.047 | 1.005 | 1.002 | 1.003 |
| *tetW*  (Tetracycline) | 1.120 | 1.025 | 1.132 | 1.025 | 1.012 | 1.003 | 1.007 | 1.025 | 1.25 |
| *qepA*  (Quinolone) | 1.001 | 1.001 | 1.005 | 1.005 | 1.006 | **1.006** | 1.220 | **1.015** | 1.001 |
| *qnrS*  (Quinolone) | 1.001 | 1.012 | 1.003 | 1.028 | 1.000 | 1.021 | 1.001 | 1.009 | 1.000 |
| *oqxA*  (Olaquindox) | 1.001 | 1.008 | 1.001 | 1.002 | 1.000 | 1.000 | 1.000 | 1.000 | 1.003 |
| *oqxB*  (Olaquindox) | 1.059 | 1.002 | 1.016 | 1.052 | 1.030 | 1.012 | 1.019 | 1.016 | 1.023 |
| *aac(6′)-Ib*  (Aminoglycoside) | 1.033 | 1.061 | 1.004 | 1.001 | 1.006 | 1.000 | 1.006 | 1.002 | 1.005 |
| *ami*  (Aminoglycoside) | 1.000 | 1.009 | 1.002 | 1.016 | 1.003 | 1.016 | 1.001 | 1.026 | 1.047 |
| *blaP*  (Beta-lactam) | 1.001 | 1.021 | 1.001 | 0.955 | 1.002 | 1.019 | 1.002 | 1.009 | 1.001 |
| *Cmr*  (Chloramphenicol) | 1.001 | 1.030 | 1.007 | 1.045 | 1.001 | 1.000 | 1.000 | 1.000 | 1.001 |

**Supplementary Table 8**. Every 7^th^ day interval antimicrobial gene expression result of *E.coli*

| **GENE** | 1^st^ Day | 7^th^  Day | 14^th^ Day | 21th Day | 28^th^  Day | 35^th^ Day | 42th Day | 49^th^ Day | 56^th^ Day |
| --- | --- | --- | --- | --- | --- | --- | --- | --- | --- |
| *sul1*  (Sulfonamide) | 1.053 | 1.086 | 1.001 | 1.008 | 1.006 | 1.013 | 1.031 | 1.001 | 1.013 |
| *sul2*  (Sulfonamide) | 1.001 | 1.025 | 1.004 | 1.003 | 1.005 | 1.004 | 1.018 | 1.087 | 1.001 |
| *tetM*  (Tetracycline) | 1.004 | 1.009 | 1.002 | 1.006 | 1.019 | 1.019 | 1.011 | 1.029 | 1.000 |
| *tetW*  (Tetracycline) | 1.005 | 1.007 | 1.003 | 1.029 | 1.033 | 1.043 | 1.011 | 1.063 | 1.001 |
| *qepA*  (Quinolone) | 1.000 | 1.001 | 1.000 | 1.001 | 1.004 | 1.002 | 1.002 | 1.003 | 1.004 |
| *qnrS*  (Quinolone) | 1.057 | 1.057 | 1.000 | 1.167 | 1.011 | 1.059 | 1.009 | 1.007 | 1.001 |
| *oqxA*  (Olaquindox) | 1.001 | 1.000 | 1.000 | 1.167 | 1.004 | 1.005 | 1.003 | 1.008 | 1.022 |
| *oqxB*  (Olaquindox) | 1.001 | 1.006 | 1.002 | 1.167 | 1.009 | 1.009 | 1.023 | 1.007 | 1.001 |
| *aac(6′)-Ib*  (Aminoglycoside) | 1.007 | 1.003 | 1.002 | 1.005 | 1.003 | 1.009 | 1.007 | 1.007 | 1.002 |
| *ami*  (Aminoglycoside) | 1.001 | 1.001 | 1.001 | 1.001 | 1.002 | 1.002 | 1.005 | 1.008 | 1.001 |
| *blaP*  (Beta-lactam) | 1.001 | 1.006 | 1.001 | 1.003 | 1.008 | 1.003 | 1.015 | 1.001 | 1.010 |
| *Cmr*  (Chloramphenicol) | 1.005 | 1.005 | 1.001 | 1.005 | 1.019 | 1.006 | 1.016 | 1.075 | 1.007 |
